# Crystal structure of (2*E*)-1-(4-hy­droxy-1-methyl-2-oxo-1,2-di­hydro­quinolin-3-yl)-3-(4-hy­droxy-3-meth­oxy­phen­yl)prop-2-en-1-one

**DOI:** 10.1107/S2056989015005630

**Published:** 2015-03-28

**Authors:** Peter Mangwala Kimpende, Ngoc Thanh Nguyen, Minh Thao Nguyen, Quoc Trung Vu, Luc Van Meervelt

**Affiliations:** a Chemistry Department, University of Kinshasa, Kinshasa XI BP 190, Democratic Republic of Congo; b Faculty of Chemical Technology, Hanoi University of Industry, Minh Khai Commune – Tu Liem District, Hanoi, Vietnam; c Faculty of Chemistry, Hanoi University of Science, 334 - Nguyen Trai – Thanh Xuan District, Hanoi, Vietnam; d Chemistry Department, Hanoi National University of Education, 136 - Xuan Thuy – Cau Giay, Hanoi, Vietnam; e Chemistry Department, KU Leuven, Celestijnenlaan 200F, B-3001 Leuven (Heverlee), Belgium

**Keywords:** crystal structure, 4-hy­droxy-1,2-di­hydro­quinolin-2(1*H*)-one, α,β-unsaturated ketones, hydrogen bonding, π–π inter­actions

## Abstract

In the title compound, C_20_H_17_NO_5_, the dihedral angle between the mean plane of the di­hydro­quinoline ring system (r.m.s. deviation = 0.003 Å) and the benzene ring is 1.83 (11)°. The almost planar conformation is a consequence of an intra­molecular O—H⋯O hydrogen bond and the *E* configuration about the central C=C bond. In the crystal structure, O—H⋯O hydrogen bonds generate chains of mol­ecules along the [10-1] direction. These chains are linked *via* π–π inter­actions [inter-centroid distances are in the range 3.6410 (16)–3.8663 (17) Å].

## Abstract 2

The crystal packing of the title compound features O—H⋯O hydrogen bonds, which form one-dimensional chains of mol­ecules further linked *via* π–π inter­actions.

## Chemical context   

The quinoline ring is an important component of bioactive heterocycles because of its diversity (Larsen *et al.*, 1996 ▸; Chen *et al.*, 2001 ▸; Roma *et al.*, 2000 ▸; Dubé *et al.*, 1998 ▸; Billker *et al.*, 1998 ▸). Many derivatives containing 4-hy­droxy-1,2-di­hydro­quinolin-2(1*H*)-one have wide applications in pharmaceuticals, such as anti­cancer (Hasegawa *et al.*, 1990 ▸), anti-inflammatory (Ukrainets *et al.*, 1996 ▸) and anti­seizure (Rowley *et al.*, 1993 ▸). Some α,β-unsaturated ketones are known to have anti­malarial, anti­bacterial and anti­fungal properties (Katritzky & Rees, 1984 ▸). The anti­cancer ability of some α,β-unsaturated ketones containing a quinoline ring has also been reported (Rezig *et al.*, 2000 ▸; Nguyen, 2007 ▸). A number of the α,β-unsaturated ketones containing quinoline synthesized by the Claisen–Schmidt reaction have been reported to inhibit anti­malarial activity (Domínguez *et al.*, 2001 ▸). Moussaoui *et al.* (2002 ▸) also described the synthesis of α,β-unsaturated ketones containing a quinoline ring and claimed cytotoxicity with human leukemia cells. Here we present the synthesis and crystal structure of an α,β-unsaturated ketone derived from 3-acetyl-4-hy­droxy-*N*-methyl­quinolin-2(1*H*)-one and 4-hy­droxy-3-meth­oxy­benzaldehyde.
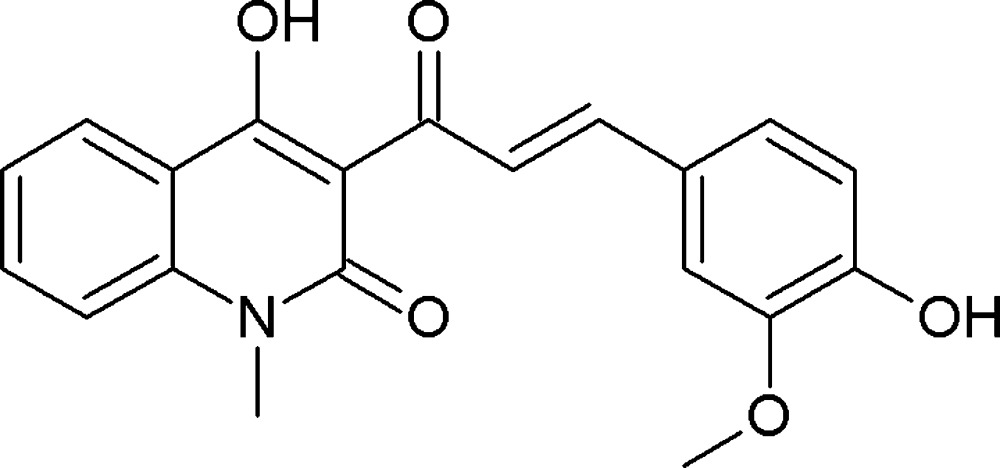


## Structural Commentary   

**Figure 1:**
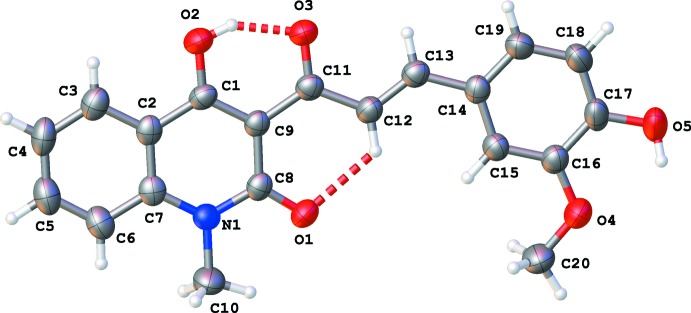
The mol­ecular structure of the title compound, with displacement ellipsoids drawn at the 50% probability level. Hydrogen bonds are shown as dashed lines (see Table 1 ▸ for details).

The mol­ecular structure of the title compound is illustrated in Fig. 1 ▸. The whole mol­ecule is almost planar with a maximum deviation from the best plane through all atoms of 0.147 (3) Å for atom C20. The di­hydro­quinoline and benzene rings make a dihedral angle of 1.83 (11)° between the best planes. The configuration of the C12=C13 bond is *E*, with a C9—C11—C12—C13 torsion angle of 177.0 (2)°. In addition, intra­molecular O2—H2⋯O3 and C12—H12⋯O1 hydrogen bonds assure the observed planarity of the structure (Table 1 ▸). Three short intra­molecular contacts are observed: H10*B*⋯O1 (2.18 Å), H5*A*⋯O4 (2.25 Å) and H13⋯O3 (2.37 Å).

## Supra­molecular features   

**Figure 2:**
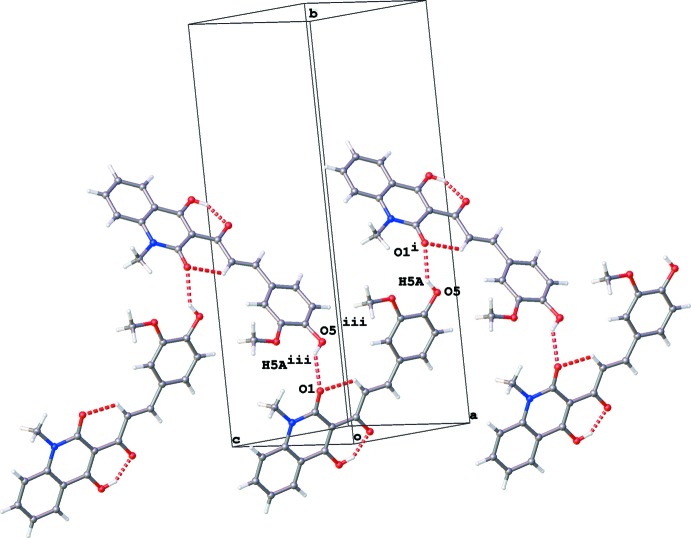
Infinite chains in the [10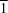] direction formed by O5—H5*A*⋯O1 hydrogen bonds (shown as red dashed lines). [Symmetry codes: (i) *x* + 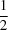, −*y* + 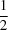, *z* − 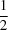; (iii) *x* − 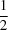, −*y* + 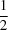, *z* + 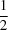.]

**Figure 3:**
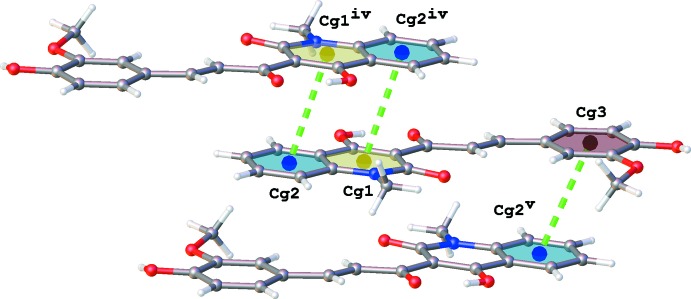
π–π inter­actions in the crystal of the title compound shown as green dashed lines. [Symmetry codes: (iv) −*x* + 1, −*y*, −*z* + 2; (v) −*x* + 2, −*y*, −*z* + 2.]

In the crystal, mol­ecules are connected *via* O5—H5*A*⋯O1 hydrogen bonds, forming chains propagating along [10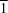] (Fig. 2 ▸ and Table 1 ▸). These chains are linked by π–π inter­actions involving both ring systems (Fig. 3 ▸) and C—H⋯O inter­actions (Table 1 ▸). The inter-centroid distances are 3.6410 (16) and 3.8663 (17) Å for π–π inter­actions involving *Cg*1⋯*Cg*2^iv^ and *Cg*3⋯*Cg*2^v^, respectively, where *Cg*1, *Cg*2 and *Cg*3 are the centroids of the N1/C1–C2/C7–C9, C2–C7 and C14–C19 rings, respectively [symmetry codes: (iv) −*x* + 1, −*y*, −*z* + 2; (v) −*x* + 2, −*y*, −*z* + 2].

## Database survey   

A search of the Cambridge Structural Database (Version 5.36; last update November 2014; Groom & Allen, 2014 ▸) for α,β-unsaturated ketones C—CH=CH—C(=O)—O gave 1281 hits of which the majority adopts an *E* configuration (C—C=C—C torsion angle around 180°) as in the title compound. For only 19 entries this torsion angle is centered around 0°. A search for 1,2-di­hydro­quinoline derivatives gave 706 hits of which none contains an α,β-unsaturated ketone at the 3-position. The angle between the best planes through the two six-membered rings in these 1,2-di­hydro­quinoline derivatives is in the range of 0–22.13°. In the title compound, this angle is 1.49 (12)°.

## Synthesis and crystallization   

The precursors 4-hy­droxy-6-methyl-2*H*-pyrano[3,2-*c*]quino­line-2,5(6*H*)-dione and 3-acetyl-4-hy­droxy-*N*-methyl­quinolin-2(1*H*)-one were prepared in high yield (87.0 and 92.5%, respectively) according to Kappe *et al.* (1994 ▸).

The title compound was synthesized by refluxing a solution of 2.17 g (0.01 mol) of 3-acetyl-4-hy­droxy-*N*-methyl­quinolin-2(1*H*)-one, 1.52 g (0.01 mol) of 4-hy­droxy-3-meth­oxy­benzaldehyde, 22 ml of chloro­form and 5 drops of piperidine (as a catalyst) in a 100 ml flask for 30 h. The precipitate was filtered off and recrystallized from ethanol to obtain the title product as yellow crystals. The yield was 2.03 g (58%); m.p. 505–506 K, *R*_f_ 0.7 (CHCl_3_–C_2_H_5_OH = 7:1 *v*/*v*).

IR (KBr, cm^−1^): 3357, 3115 (ν_OH_); 1637 (ν_C=O_); 997 (ν_CH= *trans*_). ^1^H NMR (δ p.p.m.; DMSO-*d*_6_, Bruker Avance 500 MHz): 8.47 (1H, *d*, ^2^ *J* = 16.0 Hz, H_β_), 7.92 (1H, *d*, ^2^ *J* = 16.0 Hz, H_α_), 3.59 (3H, *s* CH_3_-N), 7.33 (1H, *t*, ^3^ *J* = 8.0 Hz, C_6_-H), 7.55 (1H, *d*, ^3^ *J* = 8.0 Hz, C_5_-H), 7.81 (1H, *t*, ^3^ *J* = 8.0 Hz, C_7_-H), 8.13 (1H, *d*, ^3^ *J* = 8.0 Hz, C_8_-H), 3.85 (3H, *s*, OCH_3_), 6.89 (2H, *d*, ^3^ *J* = 8.0 Hz, C_13_-H), 7.27 (1H, *d*, ^3^ *J* = 8.0 Hz, C_12_-H), 7.30 (1H, *s*, C_9_-H), 9.89 (1H, *s*, C_4_-OH). Calculation for C_20_H_17_NO_5_: *M* = 351 au. Found (by ESI MS, *m*/*z*): 351 (*M* ^+^).

## Refinement   

Crystal data, data collection and structure refinement details are summarized in Table 2 ▸. All H atoms were refined using a riding model with stretchable C—H and O—H distances and with *U*_iso_ = 1.2*U*_eq_(C) (1.5 times for methyl and hydroxyl groups).

## Supplementary Material

Crystal structure: contains datablock(s) I. DOI: 10.1107/S2056989015005630/rz5152sup1.cif

Structure factors: contains datablock(s) I. DOI: 10.1107/S2056989015005630/rz5152Isup2.hkl

Supporting information file. DOI: 10.1107/S2056989015005630/rz5152Isup3.cml

CCDC reference: 1054894

Additional supporting information:  crystallographic information; 3D view; checkCIF report

## Figures and Tables

**Table 1:** Hydrogen-bond geometry (, )

*D*H*A*	*D*H	H*A*	*D* *A*	*D*H*A*
O2H2O3	0.84	1.65	2.407(3)	148
O5H5*A*O1^i^	0.84	2.05	2.730(3)	137
C12H12O1	0.98	2.18	2.822(3)	124
C10H10*C*O3^ii^	0.98	2.56	3.523(3)	167

Symmetry codes: (i) 
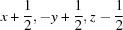
; (ii) 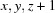.

**Table 2:** Experimental details

Crystal data	
Chemical formula	C_20_H_17_NO_5_
*M*_r_	351.35
Crystal system, space group	Monoclinic, *P*2_1_/*n*
Temperature (K)	100
*a*, *b*, *c* ()	8.3634(8), 22.664(2), 8.8079(9)
()	95.413(3)
*V* (^3^)	1662.1(3)
*Z*	4
Radiation type	Cu *K*
(mm^1^)	0.84
Crystal size (mm)	0.58 0.22 0.04

Data collection	
Diffractometer	Bruker SMART 6000
Absorption correction	Multi-scan (*SADABS*; Bruker, 2003 ▸)
*T*_min_, *T*_max_	0.641, 0.967
No. of measured, independent and observed [*I* > 2(*I*)] reflections	15707, 2881, 1889
*R*_int_	0.086
(sin /)_max_ (^1^)	0.595

Refinement	
*R*[*F* ^2^ > 2(*F* ^2^)], *wR*(*F* ^2^), *S*	0.056, 0.156, 1.02
No. of reflections	2881
No. of parameters	239
H-atom treatment	H-atom parameters constrained
_max_, _min_ (e ^3^)	0.23, 0.19

Computer programs: *SMART* and *SAINT* (Bruker, 2003 ▸), *SHELXS97* and *SHELXL97* (Sheldrick, 2008 ▸) and *OLEX2* (Dolomanov *et al.*, 2009 ▸).

## supplementary crystallographic information

### Crystal data

**Table:**

C_20_H_17_NO_5_	*Z* = 4
*M**_r_* = 351.35	*F*(000) = 736
Monoclinic, *P*2_1_/*n*	*D*_x_ = 1.404 Mg m^−^^3^
*a* = 8.3634 (8) Å	Cu *K*α radiation, λ = 1.54178 Å
*b* = 22.664 (2) Å	µ = 0.84 mm^−^^1^
*c* = 8.8079 (9) Å	*T* = 100 K
β = 95.413 (3)°	Block, yellow
*V* = 1662.1 (3) Å^3^	0.58 × 0.22 × 0.04 mm

### Data collection

**Table:**

Bruker SMART 6000 diffractometer	2881 independent reflections
Radiation source: fine-focus sealed tube	1889 reflections with *I* > 2σ(*I*)
Crossed Gοbel mirrors monochromator	*R*_int_ = 0.086
w\ and φ scans	θ_max_ = 66.6°, θ_min_ = 3.9°
Absorption correction: multi-scan (*SADABS*; Bruker, 2003)	*h* = −9→9
*T*_min_ = 0.641, *T*_max_ = 0.967	*k* = −26→26
15707 measured reflections	*l* = −10→10

### Refinement

**Table:**

Refinement on *F*^2^	Primary atom site location: structure-invariant direct methods
Least-squares matrix: full	Secondary atom site location: difference Fourier map
*R*[*F*^2^ > 2σ(*F*^2^)] = 0.056	Hydrogen site location: inferred from neighbouring sites
*wR*(*F*^2^) = 0.156	H-atom parameters constrained
*S* = 1.02	*w* = 1/[σ^2^(*F*_o_^2^) + (0.0641*P*)^2^ + 0.0033*P*] where *P* = (*F*_o_^2^ + 2*F*_c_^2^)/3
2881 reflections	(Δ/σ)_max_ < 0.001
239 parameters	Δρ_max_ = 0.23 e Å^−^^3^
0 restraints	Δρ_min_ = −0.19 e Å^−^^3^

### Special details

**Table:**

Geometry. All e.s.d.'s (except the e.s.d. in the dihedral angle between two l.s. planes) are estimated using the full covariance matrix. The cell e.s.d.'s are taken into account individually in the estimation of e.s.d.'s in distances, angles and torsion angles; correlations between e.s.d.'s in cell parameters are only used when they are defined by crystal symmetry. An approximate (isotropic) treatment of cell e.s.d.'s is used for estimating e.s.d.'s involving l.s. planes.
Refinement. Refinement of *F*^2^ against ALL reflections. The weighted *R*-factor *wR* and goodness of fit *S* are based on *F*^2^, conventional *R*-factors *R* are based on *F*, with *F* set to zero for negative *F*^2^. The threshold expression of *F*^2^ > σ(*F*^2^) is used only for calculating *R*-factors(gt) *etc*. and is not relevant to the choice of reflections for refinement. *R*-factors based on *F*^2^ are statistically about twice as large as those based on *F*, and *R*- factors based on ALL data will be even larger.

### Fractional atomic coordinates and isotropic or equivalent isotropic displacement parameters (Å^2^)

**Table:**

	*x*	*y*	*z*	*U*_iso_*/*U*_eq_	
C1	0.7024 (3)	−0.04442 (11)	0.9256 (3)	0.0415 (6)	
N1	0.7229 (2)	0.02301 (9)	1.1896 (2)	0.0420 (5)	
O1	0.8821 (2)	0.08925 (8)	1.0865 (2)	0.0512 (5)	
C2	0.6258 (3)	−0.06452 (11)	1.0557 (3)	0.0413 (6)	
O2	0.6850 (2)	−0.07762 (9)	0.8038 (2)	0.0583 (6)	
H2	0.7380	−0.0633	0.7362	0.088*	
C3	0.5393 (3)	−0.11726 (12)	1.0516 (4)	0.0523 (7)	
H3	0.5284	−0.1402	0.9609	0.063*	
O3	0.8460 (2)	−0.00808 (8)	0.6787 (2)	0.0546 (5)	
C4	0.4692 (3)	−0.13626 (13)	1.1794 (4)	0.0587 (8)	
H4	0.4108	−0.1723	1.1772	0.070*	
O4	1.2629 (3)	0.28691 (9)	0.7891 (2)	0.0675 (6)	
C5	0.4853 (3)	−0.10230 (14)	1.3095 (4)	0.0620 (8)	
H5	0.4376	−0.1152	1.3973	0.074*	
O5	1.3629 (3)	0.29415 (9)	0.5092 (3)	0.0723 (7)	
H5A	1.3606	0.3205	0.5759	0.109*	
C6	0.5689 (3)	−0.05015 (13)	1.3150 (3)	0.0540 (7)	
H6	0.5789	−0.0277	1.4064	0.065*	
C7	0.6394 (3)	−0.02989 (11)	1.1873 (3)	0.0421 (6)	
C8	0.8029 (3)	0.04333 (11)	1.0694 (3)	0.0394 (6)	
C9	0.7893 (3)	0.00803 (10)	0.9302 (3)	0.0370 (5)	
C10	0.7290 (4)	0.06001 (14)	1.3261 (3)	0.0635 (8)	
H10A	0.6203	0.0733	1.3420	0.095*	
H10B	0.7974	0.0944	1.3128	0.095*	
H10C	0.7732	0.0372	1.4148	0.095*	
C11	0.8604 (3)	0.02611 (11)	0.7932 (3)	0.0418 (6)	
C12	0.9447 (3)	0.08179 (11)	0.7778 (3)	0.0447 (6)	
H12	0.9507	0.1094	0.8594	0.054*	
C13	1.0134 (3)	0.09468 (11)	0.6516 (3)	0.0429 (6)	
H13	1.0054	0.0654	0.5741	0.051*	
C14	1.0990 (3)	0.14816 (11)	0.6180 (3)	0.0420 (6)	
C15	1.1320 (3)	0.19255 (11)	0.7263 (3)	0.0447 (6)	
H15	1.0944	0.1890	0.8244	0.054*	
C16	1.2192 (3)	0.24159 (12)	0.6913 (3)	0.0480 (7)	
C17	1.2722 (3)	0.24754 (12)	0.5466 (4)	0.0536 (7)	
C18	1.2366 (4)	0.20473 (12)	0.4387 (4)	0.0584 (8)	
H18	1.2714	0.2090	0.3396	0.070*	
C19	1.1502 (3)	0.15542 (12)	0.4737 (3)	0.0524 (7)	
H19	1.1256	0.1261	0.3981	0.063*	
C20	1.2328 (4)	0.28030 (14)	0.9429 (4)	0.0699 (9)	
H20A	1.1166	0.2782	0.9499	0.105*	
H20B	1.2773	0.3141	1.0019	0.105*	
H20C	1.2834	0.2439	0.9839	0.105*	

### Atomic displacement parameters (Å^2^)

**Table:**

	*U*^11^	*U*^22^	*U*^33^	*U*^12^	*U*^13^	*U*^23^
C1	0.0403 (13)	0.0349 (13)	0.0493 (15)	0.0023 (10)	0.0045 (11)	−0.0069 (11)
N1	0.0485 (11)	0.0400 (12)	0.0372 (12)	0.0021 (9)	0.0034 (10)	0.0010 (8)
O1	0.0626 (11)	0.0393 (11)	0.0518 (11)	−0.0104 (9)	0.0065 (9)	−0.0053 (8)
C2	0.0355 (12)	0.0364 (14)	0.0522 (16)	0.0059 (10)	0.0046 (11)	0.0078 (11)
O2	0.0723 (13)	0.0472 (12)	0.0584 (13)	−0.0144 (10)	0.0210 (10)	−0.0155 (9)
C3	0.0465 (15)	0.0426 (16)	0.0679 (19)	−0.0003 (12)	0.0057 (14)	0.0037 (13)
O3	0.0706 (12)	0.0439 (11)	0.0515 (11)	−0.0091 (9)	0.0171 (10)	−0.0107 (8)
C4	0.0519 (16)	0.0476 (17)	0.077 (2)	−0.0073 (13)	0.0092 (15)	0.0175 (15)
O4	0.0914 (15)	0.0506 (13)	0.0635 (14)	−0.0232 (11)	0.0223 (12)	−0.0085 (10)
C5	0.0574 (18)	0.066 (2)	0.064 (2)	−0.0045 (15)	0.0134 (16)	0.0199 (15)
O5	0.0931 (16)	0.0444 (13)	0.0869 (18)	−0.0117 (11)	0.0471 (14)	0.0035 (10)
C6	0.0532 (15)	0.0613 (19)	0.0482 (16)	0.0000 (13)	0.0084 (13)	0.0105 (13)
C7	0.0345 (12)	0.0436 (15)	0.0476 (15)	0.0056 (10)	0.0016 (11)	0.0069 (11)
C8	0.0368 (12)	0.0336 (13)	0.0475 (15)	0.0028 (10)	0.0024 (11)	0.0029 (10)
C9	0.0355 (12)	0.0315 (13)	0.0443 (14)	0.0040 (10)	0.0051 (11)	0.0013 (10)
C10	0.086 (2)	0.064 (2)	0.0411 (16)	−0.0142 (17)	0.0089 (16)	−0.0072 (13)
C11	0.0407 (13)	0.0374 (14)	0.0479 (15)	0.0050 (11)	0.0068 (12)	−0.0028 (11)
C12	0.0455 (14)	0.0382 (14)	0.0519 (16)	−0.0020 (11)	0.0126 (12)	−0.0035 (11)
C13	0.0425 (13)	0.0378 (14)	0.0486 (15)	0.0030 (11)	0.0056 (12)	−0.0025 (11)
C14	0.0386 (12)	0.0382 (14)	0.0506 (16)	0.0026 (10)	0.0108 (12)	0.0028 (11)
C15	0.0451 (14)	0.0435 (15)	0.0470 (15)	−0.0019 (11)	0.0120 (12)	−0.0001 (11)
C16	0.0495 (15)	0.0400 (15)	0.0564 (17)	0.0009 (11)	0.0141 (13)	−0.0009 (12)
C17	0.0584 (17)	0.0380 (15)	0.068 (2)	0.0030 (12)	0.0270 (15)	0.0101 (13)
C18	0.077 (2)	0.0479 (17)	0.0552 (18)	0.0014 (14)	0.0308 (16)	0.0037 (13)
C19	0.0632 (17)	0.0456 (17)	0.0508 (17)	0.0012 (13)	0.0185 (14)	−0.0033 (12)
C20	0.089 (2)	0.063 (2)	0.059 (2)	−0.0198 (18)	0.0139 (18)	−0.0138 (15)

### Geometric parameters (Å, º)

**Table:**

C1—C2	1.439 (4)	C8—C9	1.459 (3)
C1—O2	1.307 (3)	C9—C11	1.454 (3)
C1—C9	1.392 (3)	C10—H10A	0.9800
N1—C7	1.387 (3)	C10—H10B	0.9800
N1—C8	1.384 (3)	C10—H10C	0.9800
N1—C10	1.462 (3)	C11—C12	1.458 (3)
O1—C8	1.235 (3)	C12—H12	0.9500
C2—C3	1.396 (4)	C12—C13	1.331 (4)
C2—C7	1.395 (4)	C13—H13	0.9500
O2—H2	0.8400	C13—C14	1.452 (3)
C3—H3	0.9500	C14—C15	1.396 (4)
C3—C4	1.386 (4)	C14—C19	1.389 (4)
O3—C11	1.269 (3)	C15—H15	0.9500
C4—H4	0.9500	C15—C16	1.380 (4)
C4—C5	1.377 (4)	C16—C17	1.395 (4)
O4—C16	1.367 (3)	C17—C18	1.371 (4)
O4—C20	1.409 (4)	C18—H18	0.9500
C5—H5	0.9500	C18—C19	1.381 (4)
C5—C6	1.372 (4)	C19—H19	0.9500
O5—H5A	0.8400	C20—H20A	0.9800
O5—C17	1.359 (3)	C20—H20B	0.9800
C6—H6	0.9500	C20—H20C	0.9800
C6—C7	1.396 (4)		
			
O2—C1—C2	116.6 (2)	H10A—C10—H10B	109.5
O2—C1—C9	122.2 (2)	H10A—C10—H10C	109.5
C9—C1—C2	121.2 (2)	H10B—C10—H10C	109.5
C7—N1—C10	119.1 (2)	O3—C11—C9	118.2 (2)
C8—N1—C7	123.7 (2)	O3—C11—C12	117.7 (2)
C8—N1—C10	117.2 (2)	C9—C11—C12	124.1 (2)
C3—C2—C1	121.3 (3)	C11—C12—H12	119.4
C7—C2—C1	118.4 (2)	C13—C12—C11	121.2 (2)
C7—C2—C3	120.3 (2)	C13—C12—H12	119.4
C1—O2—H2	109.5	C12—C13—H13	116.1
C2—C3—H3	119.9	C12—C13—C14	127.8 (2)
C4—C3—C2	120.2 (3)	C14—C13—H13	116.1
C4—C3—H3	119.9	C15—C14—C13	122.1 (2)
C3—C4—H4	120.4	C19—C14—C13	119.1 (2)
C5—C4—C3	119.1 (3)	C19—C14—C15	118.8 (2)
C5—C4—H4	120.4	C14—C15—H15	119.9
C16—O4—C20	117.7 (2)	C16—C15—C14	120.2 (3)
C4—C5—H5	119.3	C16—C15—H15	119.9
C6—C5—C4	121.4 (3)	O4—C16—C15	125.4 (3)
C6—C5—H5	119.3	O4—C16—C17	114.5 (2)
C17—O5—H5A	109.5	C15—C16—C17	120.1 (3)
C5—C6—H6	119.7	O5—C17—C16	122.0 (3)
C5—C6—C7	120.5 (3)	O5—C17—C18	118.1 (3)
C7—C6—H6	119.7	C18—C17—C16	119.9 (3)
N1—C7—C2	120.0 (2)	C17—C18—H18	120.0
N1—C7—C6	121.5 (3)	C17—C18—C19	120.1 (3)
C2—C7—C6	118.5 (3)	C19—C18—H18	120.0
N1—C8—C9	117.1 (2)	C14—C19—H19	119.6
O1—C8—N1	118.6 (2)	C18—C19—C14	120.9 (3)
O1—C8—C9	124.3 (2)	C18—C19—H19	119.6
C1—C9—C8	119.5 (2)	O4—C20—H20A	109.5
C1—C9—C11	118.0 (2)	O4—C20—H20B	109.5
C11—C9—C8	122.5 (2)	O4—C20—H20C	109.5
N1—C10—H10A	109.5	H20A—C20—H20B	109.5
N1—C10—H10B	109.5	H20A—C20—H20C	109.5
N1—C10—H10C	109.5	H20B—C20—H20C	109.5
			
C1—C2—C3—C4	178.7 (2)	C7—C2—C3—C4	−1.4 (4)
C1—C2—C7—N1	1.1 (3)	C8—N1—C7—C2	−3.3 (4)
C1—C2—C7—C6	−178.3 (2)	C8—N1—C7—C6	176.0 (2)
C1—C9—C11—O3	−3.2 (3)	C8—C9—C11—O3	178.2 (2)
C1—C9—C11—C12	175.5 (2)	C8—C9—C11—C12	−3.1 (4)
N1—C8—C9—C1	−1.9 (3)	C9—C1—C2—C3	−179.7 (2)
N1—C8—C9—C11	176.7 (2)	C9—C1—C2—C7	0.5 (3)
O1—C8—C9—C1	177.2 (2)	C9—C11—C12—C13	177.0 (2)
O1—C8—C9—C11	−4.2 (4)	C10—N1—C7—C2	177.0 (2)
C2—C1—C9—C8	−0.1 (3)	C10—N1—C7—C6	−3.7 (4)
C2—C1—C9—C11	−178.7 (2)	C10—N1—C8—O1	4.2 (3)
C2—C3—C4—C5	0.4 (4)	C10—N1—C8—C9	−176.6 (2)
O2—C1—C2—C3	0.8 (4)	C11—C12—C13—C14	179.0 (2)
O2—C1—C2—C7	−179.0 (2)	C12—C13—C14—C15	5.8 (4)
O2—C1—C9—C8	179.5 (2)	C12—C13—C14—C19	−174.4 (3)
O2—C1—C9—C11	0.8 (4)	C13—C14—C15—C16	177.5 (2)
C3—C2—C7—N1	−178.8 (2)	C13—C14—C19—C18	−177.9 (3)
C3—C2—C7—C6	1.9 (4)	C14—C15—C16—O4	−178.0 (2)
C3—C4—C5—C6	0.1 (5)	C14—C15—C16—C17	1.2 (4)
O3—C11—C12—C13	−4.3 (4)	C15—C14—C19—C18	2.0 (4)
C4—C5—C6—C7	0.4 (4)	C15—C16—C17—O5	−177.8 (3)
O4—C16—C17—O5	1.5 (4)	C15—C16—C17—C18	0.4 (4)
O4—C16—C17—C18	179.7 (3)	C16—C17—C18—C19	−0.9 (5)
C5—C6—C7—N1	179.3 (2)	C17—C18—C19—C14	−0.4 (5)
C5—C6—C7—C2	−1.4 (4)	C19—C14—C15—C16	−2.4 (4)
O5—C17—C18—C19	177.4 (3)	C20—O4—C16—C15	7.6 (4)
C7—N1—C8—O1	−175.6 (2)	C20—O4—C16—C17	−171.6 (3)
C7—N1—C8—C9	3.6 (3)		

### Hydrogen-bond geometry (Å, º)

**Table:**

*D*—H···*A*	*D*—H	H···*A*	*D*···*A*	*D*—H···*A*
O2—H2···O3	0.84	1.65	2.407 (3)	148
O5—H5*A*···O1^i^	0.84	2.05	2.730 (3)	137
C12—H12···O1	0.98	2.18	2.822 (3)	124
C10—H10*C*···O3^ii^	0.98	2.56	3.523 (3)	167

Symmetry codes: (i) *x*+1/2, −*y*+1/2, *z*−1/2; (ii) *x*, *y*, *z*+1.
